# Case report: Use of ^68^Ga-DOTATATE-PET for treatment guidance in complex meningioma disease

**DOI:** 10.3389/fonc.2022.1017339

**Published:** 2022-10-12

**Authors:** Anna-Katharina Meißner, Niklas von Spreckelsen, Abdulkader Al Shughri, Anna Brunn, Gina Fuertjes, Marc Schlamann, Matthias Schmidt, Markus Dietlein, Daniel Rueß, Maximilian I. Ruge, Norbert Galldiks, Roland Goldbrunner

**Affiliations:** ^1^ Department of General Neurosurgery, Center for Neurosurgery, Faculty of Medicine and University Hospital Cologne, University of Cologne, Cologne, Germany; ^2^ Department of Neuropathology, Faculty of Medicine and University Hospital Cologne, University of Cologne, Cologne, Germany; ^3^ Department of Diagnostic and Interventional Radiology, Faculty of Medicine and University Hospital Cologne, University of Cologne, Cologne, Germany; ^4^ Center for Integrated Oncology (CIO), Universities of Aachen, Bonn, Cologne and, Duesseldorf, Germany; ^5^ Department of Nuclear Medicine, Faculty of Medicine and University Hospital Cologne, University of Cologne, Cologne, Germany; ^6^ Department of Stereotaxy and Functional Neurosurgery, Center for Neurosurgery, Faculty of Medicine and University Hospital Cologne, University of Cologne, Cologne, Germany; ^7^ Department of Neurology, Faculty of Medicine and University Hospital Cologne, University of Cologne, Cologne, Germany; ^8^ Institute of Neuroscience and Medicine (INM-3), Research Center Juelich, Juelich, Germany

**Keywords:** complex meningioma disease, atypical meningioma, 68 Ga-DOTATATE-PET, SSTR2 expression, case report

## Abstract

Currently, contrast-enhanced MRI is the method of choice for treatment planning and follow-up in patients with meningioma. However, positron emission tomography (PET) imaging of somatostatin receptor subtype 2 (SSTR2) expression using ^68^Ga-DOTATATE may provide a higher sensitivity for meningioma detection, especially in cases with complex anatomy or in the recurrent setting. Here, we report on a patient with a multilocal recurrent atypical meningioma, in which ^68^Ga-DOTATATE PET was considerably helpful for treatment guidance and decision-making.

## Introduction

Meningiomas display a high expression of somatostatin receptor subtype 2 (SSTR2), which was shown to be a very sensitive and highly specific biomarker to distinguish meningiomas from healthy brain tissue independent of histological subtype and WHO grade (1, 2). SSTR2 has also been shown to be a prognostic factor and a promising therapeutic target in other tumor entities such as gliomas and neuroendocrine tumors (3–5). The expression of SSTR2 enables the use of functional imaging with somatostatin receptor ligands, such as ^68^Ga-DOTATATE (6, 7). Positron emission tomography (PET) using SSTR2 ligands was found to show a higher contrast between meningiomas and surrounding tissues compared to conventional contrast-enhanced MRI, leading to improved sensitivity in meningioma detection (8). Regarding current guidelines, MRI is the method of choice for meningioma diagnostics worldwide (9). In previous studies, additional benefits of PET imaging for diagnostics, treatment planning, and treatment guidance, especially in complex and recurrent meningiomas, could be shown (10–16). These advantages were also recently highlighted by the Response Assessment in Neuro-Oncology (RANO)/PET group and the current European Association of Neuro-Oncology guideline (EANO) (9, 17).

Here, we report on a case of a patient with a complex multimodally treated recurrent atypical meningioma in which ^68^Ga-DOTATATE PET was considerably helpful for treatment guidance and decision-making.

## Case description

**Figure 1:**
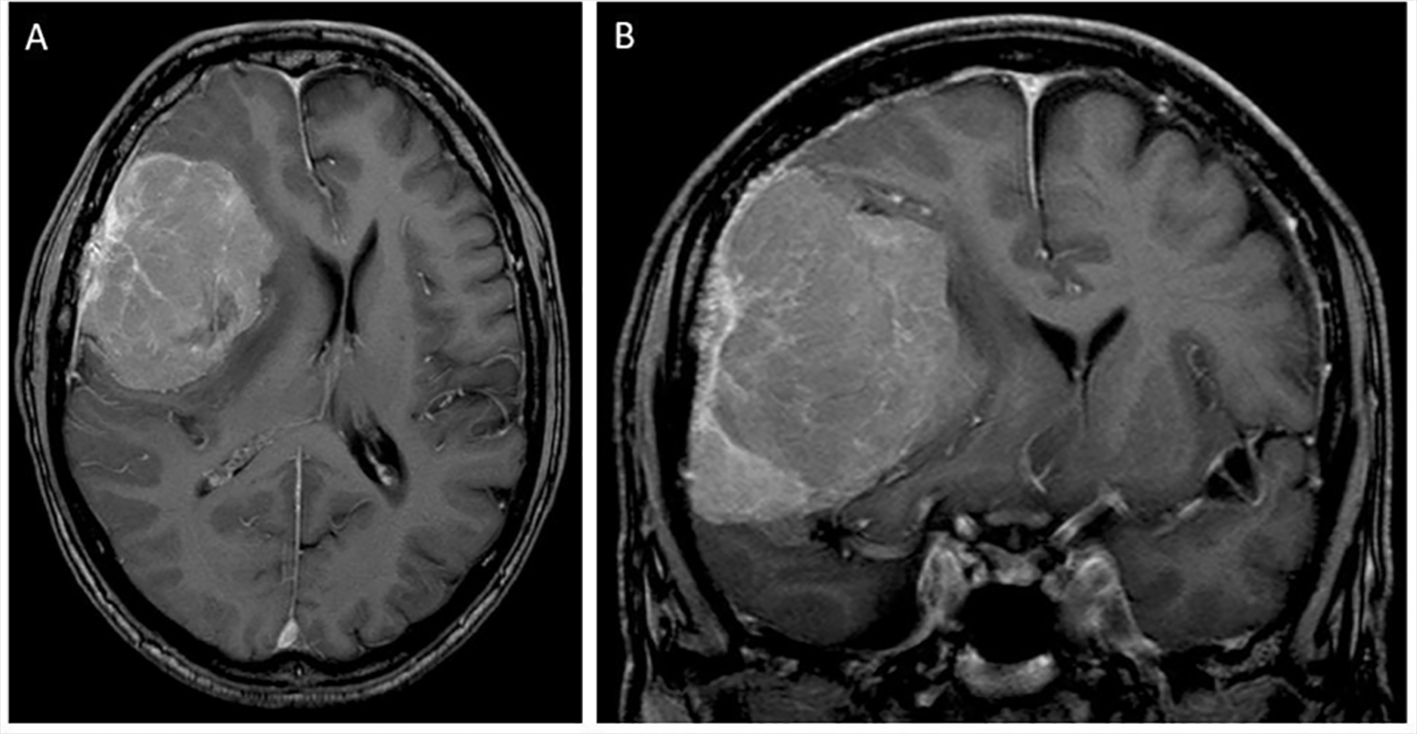
T1-contrast-enhanced MRI scan in axial **(A)** and coronal **(B)** sections at the initial diagnosis showing a large right frontal (approximately 5.5 cm × 7.0 cm) tumor with dural attachment, suggestive of a meningioma, with midline and brain shift.

A 63-year-old man presented with progressive mental alterations and mild left-sided hemiparesis. MRI revealed a large (5.5 cm × 7.0 cm) contrast-enhancing lesion in the right frontal lobe with attachment to the dura, suggestive of a meningioma, with a distinctive perifocal edema and midline shift of 11 mm (**Figure 1**). Due to the relevant mass effect and progressive symptoms, the tumor was surgically resected (Simpson Grade 1) using a right frontal approach supported by neuronavigation.

Two days after surgery, revision surgery due to a space-occupying subdural hematoma and, in the further course, a second revision surgery with removal of the bone flap for postoperative infection were necessary. The patient recovered well and was discharged with a mild left-sided hemiparesis for rehabilitation. The neuropathological analysis demonstrated an atypical meningioma WHO grade 2 according to the fifth edition of the WHO Classification of Tumors of the Central Nervous System (CNS) published in 2021. After implantation of an autologous cranioplasty 3 months later, the patient was followed up with MRI scans every 6 months according to the current guidelines (9).

**Figure 2:**
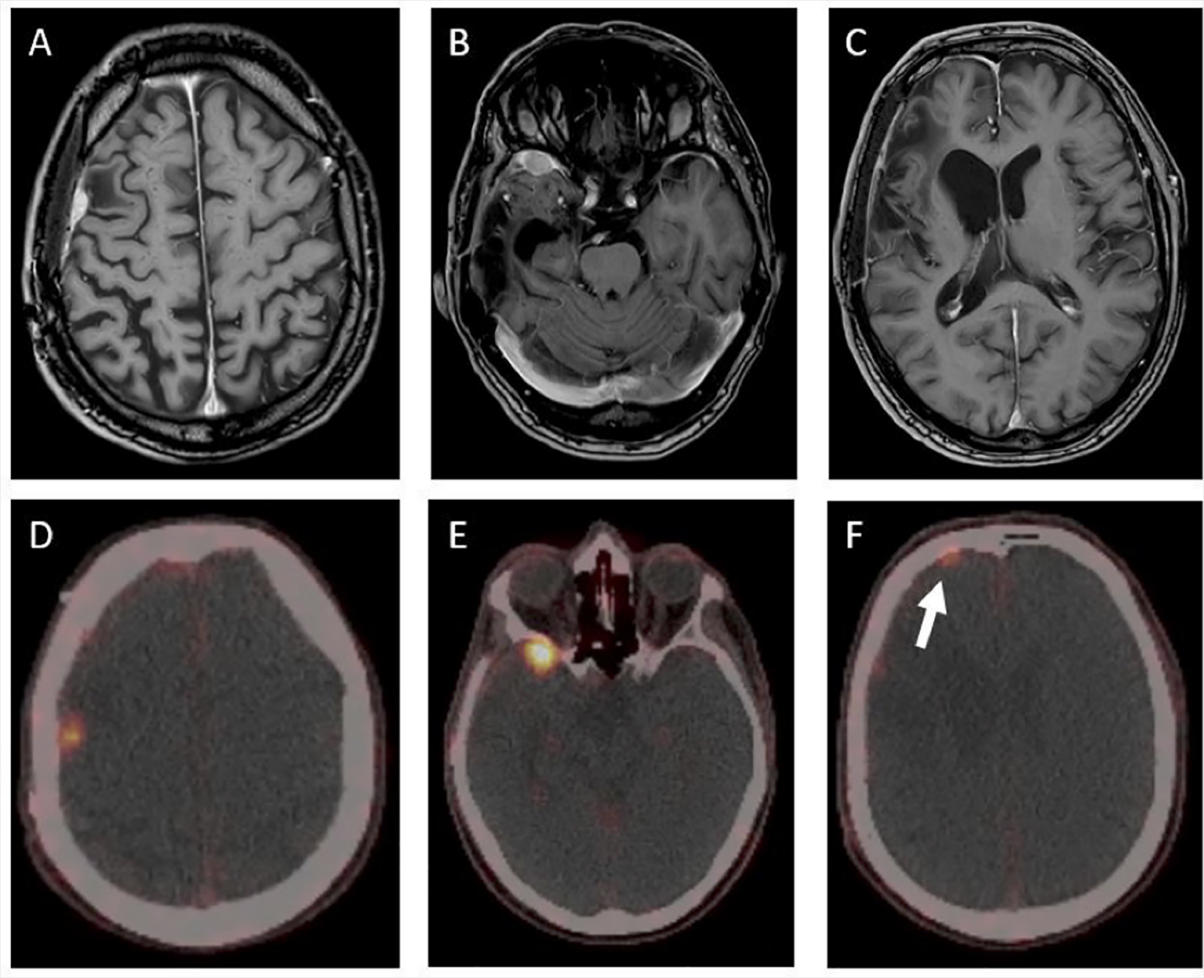
T1-contrast-enhanced MRI and ^68^Ga-DOTATATE-PET/CT showing three PET-positive lesions in different locations **(D–F)**. The contrast- enhanced MRI **(A–C)** showed no clear correlate for the displayed PET-positive right frontopolar lesion (white arrow). The maximum standardized uptake value (SUV_max_) was lower in the previously irradiated lesion **(D)** compared to those of the untreated lesions **(E, F)** (SUV_max_ of 1.9 *vs*. SUV_max_ of 9.8 and 3.0).

**Figure 3:**
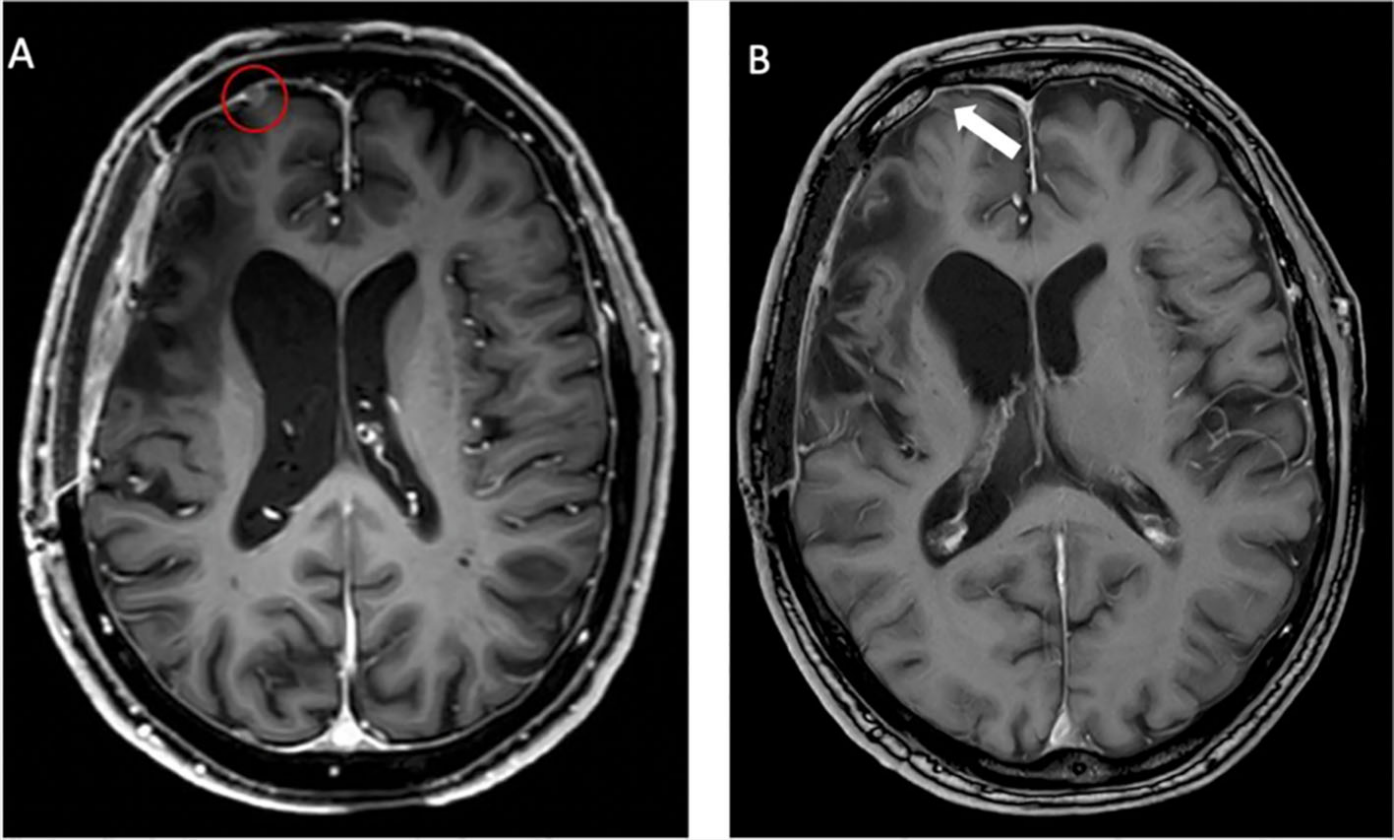
Follow-up MRI after 10 weeks **(A)** showing a progressive lesion (red circle) compared to the baseline MRI **(B)**. At initial evaluation, only a small dural alteration was observed (white arrow). In contrast, PET imaging detected considerably increased somatostatin receptor (SSTR) expression as assessed by ^68^Ga-DOTATATE uptake.

**Figure 4:**
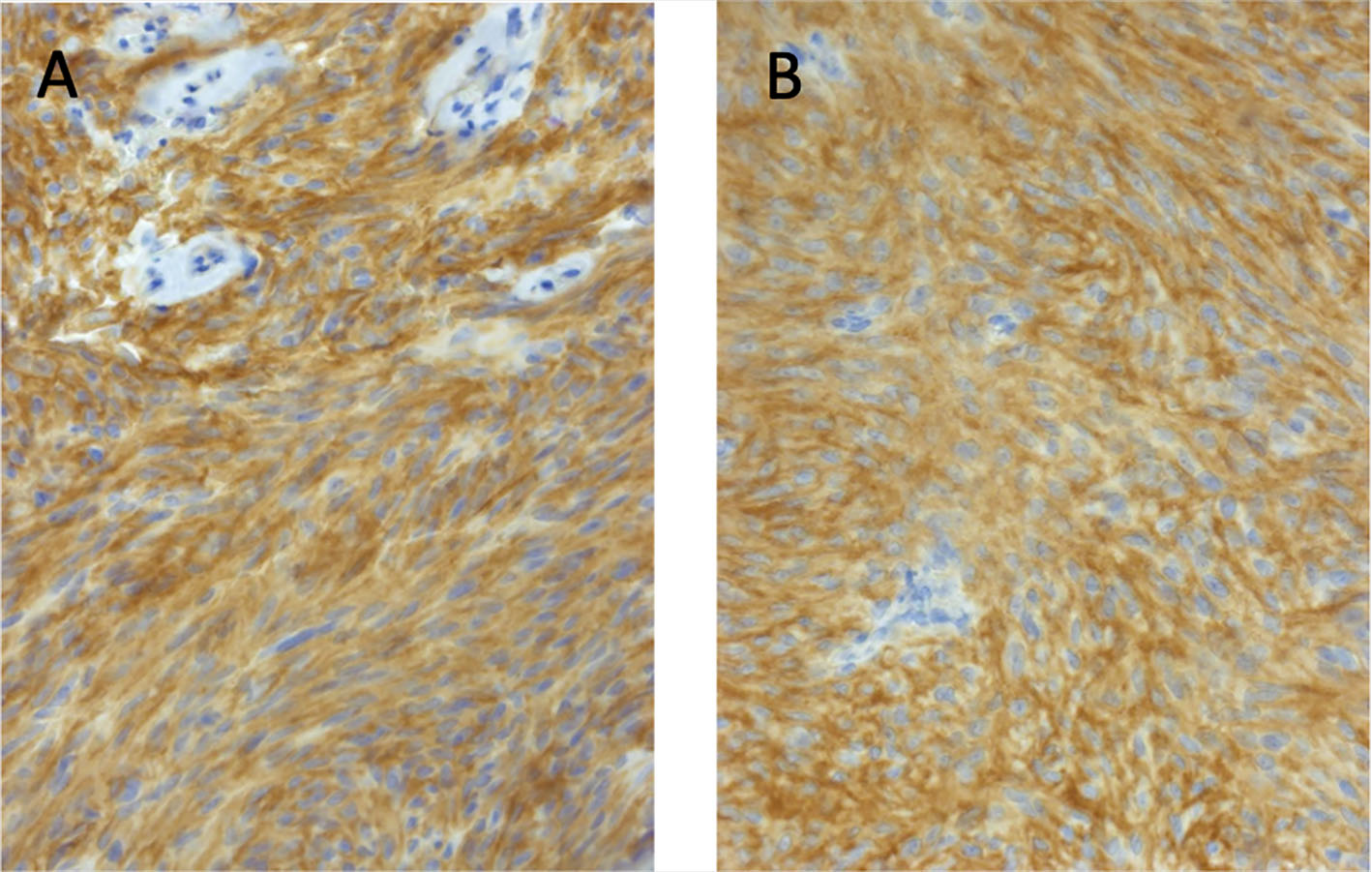
Immunohistochemistry did not reveal differences in the expression of somatostatin receptor subtype 2A (SSTR2A) between the right temporopolar **(A)** and right frontal tumor **(B)**. Immunohistochemistry with polyclonal rabbit anti-SSTR2A, slide counterstain hematoxylin (1:100 dilution; Zytomed-Systems), original magnification ×400.

Twenty-nine months after the initial surgery, a small tumor recurrence adjacent to the resection cavity was treated with robotic image-guided stereotactic radiosurgery (SRS) (16 Gy, 80% isodose) and remained stable in the following scans. Forty-eight months after initial diagnosis, follow-up MRI showed a new contrast-enhancing right temporopolar lesion, suggestive of a distant meningioma relapse. To exclude further lesions, a ^68^Ga-DOTATATE PET scan was performed, showing increased tracer uptake in the right temporopolar region and in the right frontal area of the lesion previously treated with SRS. In contrast to MRI, ^68^Ga-DOTATATE PET revealed an additional distant small dural and intraosseous right frontopolar lesion (**Figure 2**). The postoperative follow-up MRI 10 weeks later showed a progressive dural thickening and a small contrast-enhancing lesion in spatial correspondence to the PET-positive frontopolar lesion. Reviewing the baseline MRI, a small dural alteration in this particular region may have been interpreted as abnormal (**Figure 3**). After surgical resection of the right temporopolar and previously irradiated right frontal lesion, adjuvant fractionated radiotherapy (59.2 Gy; 2 × 1.6 Gy/day) including the PET-avid frontopolar area was performed. The histological report demonstrated recurrence of the atypical meningioma with a high mitotic rate in the right temporopolar tumor and proof of vital tumor cells with singular mitosis and lower proliferative rate in the previously irradiated frontal tumor. Immunohistochemistry did not reveal differences in the expression of SSTR2A between the two lesions (**Figure 4**).

The patient tolerated the surgery and radiation procedures well without further neurological and cognitive impairment. In the follow-up MRI scans every 6 months, the irradiated frontopolar lesion was stable. There was no evidence of tumor recurrence. A follow-up ^68^Ga-DOTATATE PET after 12 months in accordance with the MRI showed a favorable treatment response without any tracer uptake.

## Discussion

Complex meningiomas located at the skull base, tumors with intraosseous growth, and WHO CNS grade 2 (atypical) and 3 (malignant) meningiomas are particularly difficult to treat (9). They tend to show a more aggressive clinical behavior and higher recurrence rates (18). Frequently, multimodal therapeutic approaches with complete or partial surgical resection and subsequent radiosurgery or radiotherapy are needed (19–21). Standard imaging modalities such as CT and MRI have limitations in terms of the delineation of meningiomas with complex anatomy and the differentiation of recurrent tumors from postoperative changes such as scars (8, 22). It has been demonstrated that ^68^Ga-DOTATATE PET shows higher sensitivity and improved delineation of meningiomas, especially for those located at the skull base, adjacent to the falx cerebri, and tumors with bony infiltration, compared to conventional MRI (8, 23). Accordingly, in our case, not only the temporal recurrent and potentially viable frontal tumor was delineated using ^68^Ga-DOTATATE PET but also the small dural lesion without initial MRI correlate was detected earlier on PET, enabling a high-precision treatment before further tumor growth (22, 24). Thus, additional information on the exact tumor extent can be used for optimized surgical or radiotherapy planning in recurrent tumors, as highlighted by our case.

Regarding the quantitative correlation of SSTR2 expression and PET tracer uptake, Rachinger et al. (24) analyzed in a prospective study 115 meningioma tissue samples and were able to show a positive correlation between the immunohistochemically defined grade of SSTR2 expression and ^68^Ga-DOTATATE uptake on PET. However, there was no difference in the immunohistochemical grade of SSTR2 expression in tumor samples at initial diagnosis and in recurrent tumors. In that study, a threshold of 2.3 for the maximum standardized uptake value (SUV_max_) was able to discriminate between tumor and non-tumor tissue (sensitivity, 90%; specificity, 74%). Three of the analyzed recurrent tumors were previously irradiated. Nevertheless, no information was provided about the SUV_max_ in these cases (24). In our case, the SRS-treated tumor showed a lower SUV_max_ of 1.9 compared to the untreated lesions (SUV_max_ of 9.8 in the temporal lesion and SUV_max_ of 3.0 in the frontopolar lesion) in the PET scan 19 months after radiation, even though no difference in the SSTR2 expression was detectable by immunohistochemical analysis.

Interestingly, the follow-up ^68^Ga-DOTATATE PET 12 months after revision surgery and fractionated radiotherapy of the resection cavity and frontopolar lesion revealed no further tracer uptake in the irradiated areas. A possible reason may be the differences in the delivered radiation dose (16 Gy *vs*. 59.6 Gy), resulting in a lower biological equivalent dose of approximately 41.6 Gy for radiosurgery compared to approximately 79.3 Gy for fractionated radiotherapy. Up to now, there are no data in the literature on changes in SSTR2 expression grade and SUV_max_ in ^68^Ga-DOTATATE PET after fractional radiotherapy or radiosurgery. This will be a challenge for the near future.

The present case report suggests that ^68^Ga-DOTATATE PET is of considerable value for treatment decision-making in patients with recurrent and atypical meningiomas, especially with bony involvement, which have a high risk of recurrence. More data need to be collected regarding the correlation of SSTR2 expression and tracer uptake in previously irradiated tumors, which would allow more insight in radiation biology.

## Data availability statement

The original contributions presented in the study are included in the article/supplementary material. Further inquiries can be directed to the corresponding author.

## Ethics statement

Written informed consent was obtained from the individual(s) for the publication of any potentially identifiable images or data included in this article.

## Author contributions

Data acquisition: A-KM, RG, AB, AA, DR. Writing of manuscript drafts: AM, NG, RG, MR, DR. All authors contributed to the article and approved the submitted version.

## Conflict of interest

The authors declare that the research was conducted in the absence of any commercial or financial relationships that could be construed as a potential conflict of interest.

## Publisher’s note

All claims expressed in this article are solely those of the authors and do not necessarily represent those of their affiliated organizations, or those of the publisher, the editors and the reviewers. Any product that may be evaluated in this article, or claim that may be made by its manufacturer, is not guaranteed or endorsed by the publisher.
